# Eczema

**Published:** 1875-10

**Authors:** 


Eczema—Its Nature and Management.—In his monograph on this subject, Dr. L. D. Buckley, of New York, comes to the following conclusions :

1. True eczema is a catarrh of the skin, properly acute but very commonly subacute and chronic, and is entirely analogous to catarrh of the mucous membranes, which are, indeed, but involuted portions of the cutaneous envelope. The absence of moisture, in many cases, does not disprove this, for if the dry parts are kept covered with oiled silk or gutta-percha paper or rubber to prevent rapid evaporation, we soon find that the material forming the scales is the same as in other cases, catarrhal, which stiffens linen.

2. This catarrh tends to run a certain course, accompanied by definite pathological changes, and the disease which we meet with more commonly, is, in a large part, made up of the relics left in the skin—that is, thickening caused by a deposit of adventitious cells, mostly in the *rete mucosum*, but also capable of extending into the papillary layer, the derma, and even deep into the adipose tissue.

3. Much that is called eczema is properly only a dermatitis or ordinary inflammation of the skin, tending to spontaneous recovery when the cause is removed and irritating agencies kept away.

4. Eczema in its true sense is not a local affair, but one intimately associated with blood changes, represented in the main by a sub-oxidation or hyperacidity, as found in the stomach, kidney, etc., and in this state, moreover, is closely allied to gout, rheumatism and scrofula.

5. Debility, pure and simple, does not cause eczema, but may, by its existence, prevent either the recuperative action of nature or the beneficial effect of remedies.

6. Eczema being an acute disease, running a definite course, our treatment must consist principally in avoiding irritating elements, correcting systemic errors, debility, acidity, etc., favoring the action of the emunctories, and locally making such applications as soothe and restore tone to an irritated and inflamed integument.

7. The products of eczema, the thickening and consequent scaling and itching, are removed by stimulating applications, and by such pure tonics as act directly on the nerves, causing absorption through the capillaries, as quinine, iron, arsenic, strychnine, and the like.

8. Arsenic and zinc ointment, while serviceable in many instances, are so far from being specifics for eczema that their use is injurious in many cases, while almost always other remedies will either suffice alone or greatly assist their action.—*Pacific Medical and Surgical Journal.*

